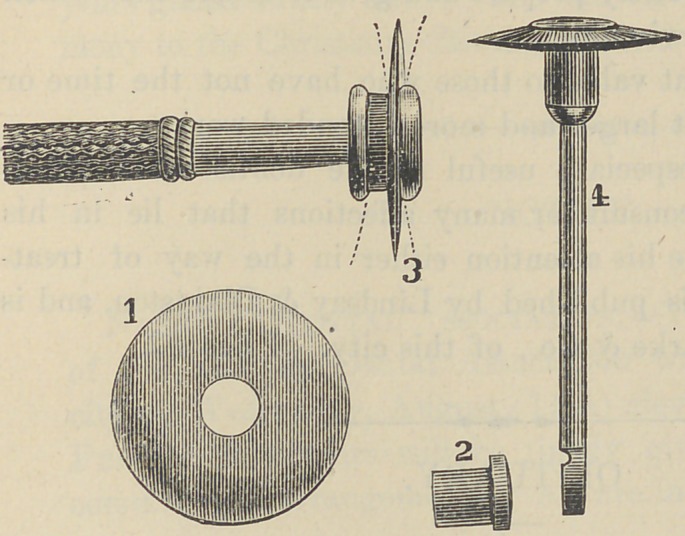# A New Appliance

**Published:** 1880-07

**Authors:** 


### A NEW APPLIANCE.

This is a "Combined shield and disk-holder," invented by Dr. M. L. S. Buckner, of Shelbyville, Ky. It consists of a small, soft rubber tube about one third of an inch in length, with a flange upon one end.

The tube is put through the disk up to the flange, then the mandril forced through the tube. This alone makes sufficient attachment for carrying the disk.

The above illustration gives at once an idea of the application and use of the holder.

No. 1 represents the disk.

No. 2 represents the shield and holder.

No. 3 represents the disk holder and hand piece.

No. 4 represents the disk mounted.

The chief advantages of this appliance are facility of mounting, ready and easy adaptation of the disk to surfaces of varying angles.

I have had this appliance in use for some time and am much pleased with it. It can be obtained at the dental depots and of the inventor.

## Figures and Tables

**Figure f1:**